# Supplementary material for: MCF7 Spheroid Development: New Insight about Spatio/Temporal Arrangements of TNTs, Amyloid Fibrils, Cell Connections, and Cellular Bridges
Source: Int J Mol Sci. 2020 Jul 29;21(15):5400. doi: 10.3390/ijms21155400 (PMC7432950; doi:10.3390/ijms21155400)
Supplement: Supplementary file 1 [file ijms-21-05400-s001.pdf]

A) Mean diameters measures (μm) at different time points

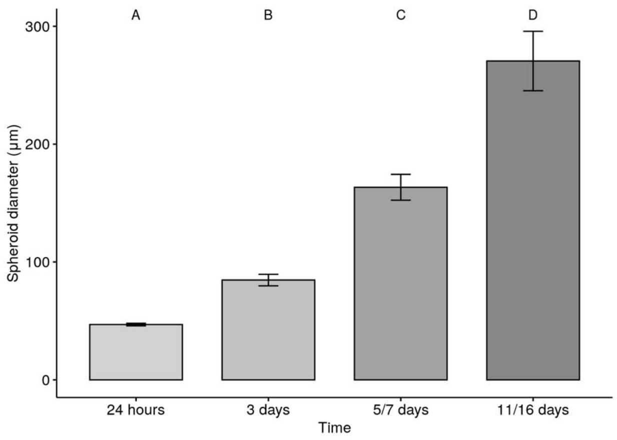

B) Mean diameters measures (μm) of untreated and NAC-treated spheroids

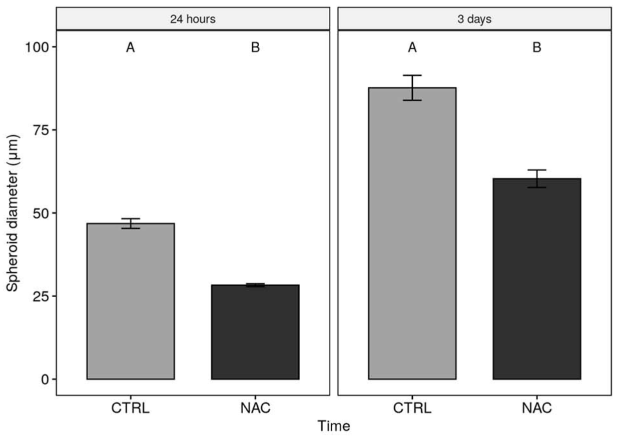

C) Mean diameters measures (μm) of untreated and NEP-treated spheroids

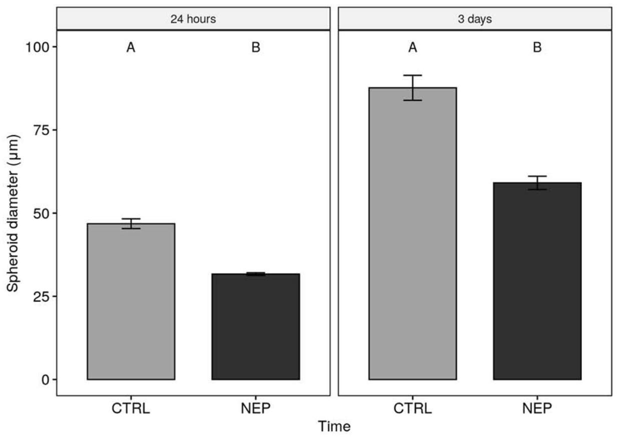

Figure S1
